# Pathology

**Published:** 1824-06-01

**Authors:** 


### Pathology.

Actio læsa suum designat singula morbum;

Preterea morbum partis mutatio signat.

---

1. *Application of the Stethoscope to the Diagnosis of Aneurisms of the Aorta.*† It is not supposed that aneurism of the aorta was recognized before the time of Vesalius; and even during the 16th century the knowledge of aortic dilatations made little progress. During the last 20 or 30 years, pathological anatomy has been so diligently cultivated that the diseases of this important vessel are now familiar to every practitioner, but the diagnosis of these diseases is not so easy a matter. It is, in fact, exceedingly difficult, by the ordinary means of examination, to distinguish between disease of the heart and disease of the aorta, unless the *latter* appears externally, when, of course, there would be no merit in the diagnosis. It is on this account that Dr. Ferrus brings forward two cases where substernal aneurism was recognized during life, without any external indication being present.

*Case* 1. Jean Hivet, 65 years of age, entered the hospital Cochin on the 28th of November, 1822. He had experienced, for three years past, palpitations of the heart and affections of the breath, which he attributed to numerous colds which he had caught. A fresh cold in Sep-

---

† Dr. Ferrus, Physician to the Saltpetriere. Archives Generales, Dec. 1823.

tember forced him to give up work, and seek refuge in an hospital. The following were the phenomena presented by the patient at the time of his entry. Complexion sallow,—heaviness about the head, with vertigo, numbness of the lower extremities—cough, with thick expectoration—anhelation on the least exercise—sense of weakness about the epigastric region—tongue red and partially coated—inappetency and nausea—irregular pulse, intermitting and not in rhythm with the action of the heart.

*Auscultation.* The respiratory murmur was heard throughout the whole circumference of the chest, and sufficiently loud. The pulsations of the heart could also be heard in every part of the chest. When the stethoscope was applied to the præcordial region, the pulsations of the ventricles were found to be very unequal, intermittent, but clear and sonorous. *Under the sternum and cartilages of the first ribs on the right side, were heard simple pulsations, accompanied by a hissing noise.* The contractions of the auricles were plainly heard towards the clavicles, but very confusedly in the region of the heart itself.

*Diagnosis.* Aneurismal hypertrophy of the ventricles—substernal aneurism of the aorta. We need not detail the prescriptions and sequel of the case. He died on the 13th of January, 1823.

*Dissection.* More than a pint of bloody serum in the left cavity of the chest. The lungs sound—mucous membrane of the trachea and bronchia red—vessels of the pericardium injected—heart, containing enormous clots of blood, one-third larger than natural—right cavities of this organ presenting nothing remarkable, except some redness of the lining membrane—left cavities dilated, especially the left ventricle, which was thrice its natural size—its parietes were much thickened—ascending arch of the aorta dilated into an aneurismal sac, the size of a man's fist—the descending aorta was indurated in its coats, but not sensibly dilated in its calibre. Its internal lining was red. There were traces of inflammation in the abdomen, both on the peritoneal and mucous membranes. In the head, the meninges were slightly thickened, and in the ventricles was contained a considerable quantity of serous effusion.

*Case* 2. Peter Pinson, 35 years of age, entered the Cochin Hospital on the 20th of May, 1823. He had experienced, for several years, a difficulty of breathing, augmented in the attempt to go up a flight of stairs, attended by palpitation of the heart. To these he paid little attention till within these three months, when the palpitation and dyspnœa became so increased, complicated with gastric derangements, that he was forced to seek hospital relief. On examination at the hospital, he presented the following phenomena:—face livid and swelled—infiltration of the lower extremities—oppression—orthopnœa—inability to procure sleep—starting from short slumbers—pulse regular, without frequency; full, vibrating in the right arm—very small in the left—cough, with viscid expectoration.

*Diagnosis by Auscultation.* The mucous rattling (râle muqueux) heard throughout every part of the chest—very strong pulsations perceptible under the sternum, and cartilages of the superior ribs on the *right side*, accompanied by a hissing noise, very distinct. This noise was also very audible in the præcordial region, covering, as it were, the pulsations of the heart. The disease was therefore pronounced to be as follows:—aneurism of the ascending aorta—hypertrophia of the heart—bronchial catarrh. Bleeding, and various means were tried, but without relief. He lingered out a wretched existence, till the 2d of July, when death put a period to his sufferings.

*Dissection.* A considerable quantity of sero-sanguineous effusion in the chest on both sides—heart thrice its natural size—aneurism of the substernal aorta the size of a child's head at birth, filling a great part of the thoracic cavity. The parietes of the left ventricle were an inch or more in thickness, and its cavity surprisingly dilated. The left auriculo-ventricular orifice was in a natural state—but that of the right side was very much enlarged, and its valves reddened, as was the lining membrane of the cavities. The lungs were greatly compressed by the enlarged heart and aorta—the mucous membrane of the trachea and bronchiæ was red, and covered with mucus. The liver and spleen were gorged with blood.

*On the pathognominic Symptoms of Aortic Aneurism.* Corvisart acknowledged that there was obscurity in these symptoms, unless the aneurismal tumour became visible or tangible. The symptoms enumerated by Corvisart, as indicating aortic dilatation, are, a wheezing noise in the respiration, a certain rustling sound heard over that part of the chest where the aneurism is situated, a dull sound emitted on percussion, smallness or irregularity of the pulse. These, it must be acknowledged, are not always present in aneurism—and are sometimes present where there is no aneurism. The only certain diagnostic symptom, therefore, according to the author of this paper is—*pulsation in the region of the aneurism itself, as heard through the stethoscope.*

---

2. *Hydrophobia.* John Brassendale, 30 years of age, was bitten by a dog, which, four days afterwards, was killed with symptoms of madness. Caustic was liberally applied to the wound, three days after the bite. On the 28th October the man shewed difficulty of swallowing, and unequivocal hydrophobia was too soon developed. Purgative glysters, laudanum in large doses, the warm bath, blisters to the chest, the muriatic acid, &c. were tried without effect.

*Dissection.* In the head nothing particular was observed, except a slight distention of the vessels of the pia mater. The mucous membrane of the larynx and trachea was red with turgid vessels. The inner surface of the œsophagus presented marks of phlogosis. In the stomach inflammation had run on almost to sphacelus. In the jejunum also were spots of a similar description.

In another case of hydrophobia which came under Dr. Bardsley's care in the Manchester Infirmary, death took place on the seventh day from the commencement of pain in the bitten part, and 56 hours after the symptoms of unequivocal hydrophobia had been developed. In this case there was turgescence of vessels about the basis of the brain, with some effusion there. No inflammation in the pharynx, larynx, or trachea. The internal membrane of the œsophagus and stomach was inflamed and studded with purple spots.

3. *Cases of Neuralgia simulating Disease of the Brain.* By Dr. MARTINET.*

Sudden and temporary attacks of *paralysis* are very embarrassing to the practitioner sometimes. We so generally associate the idea of paralysis with pressure on the brain or spinal marrow, that we usually take a gloomy view of such cases; and yet they are not always dependent on cerebral disease—at least that kind of disease which produces apoplexy and regular paralysis. Rheumatism, affections of the digestive organs, and even mental emotions, seem occasionally to determine these paralytic seizures.

*Case* 1. A woman, 60 years of age, enjoying good health previously, and leading a sedentary life, was taken all at once, on the 1st of June, 1822, with considerable loss of power in the right lower extremity, attended by a pain running in the direction of the sciatic nerve. On the 10th of the same month she complained of dull pain and sense of formication in the lumbar region of the same side. On the 12th, she felt darting pains radiating along the temple, forehead, and superior palpebra of the same side. On the 15th there was drawing of the mouth to the left, while the inferior jaw was also distorted from its usual apposition to the upper. On the 16th there was embarrassment of speech, the mouth being still more drawn to one side, with superficial pain, at intervals, in the facial nerves of that side. There was no febrile movement in the system; but the appetite was impaired. An emetic of ipecacuan was exhibited, and operated well, but produced no effect on the complaint. On the 17th the mouth still drawn—difficulty and pain in moving the right thigh—painful dartings along the scalp, and deep-seated pain in the right side of the head. These symptoms continued unchanged for three days. 21st. Tongue coated; uneasiness at the epigastrium; the other symptoms still persisting. Ten leeches to the anus—purgation by sulphate of soda. 22d. Mouth less drawn—speech more distinct—diminution of hemicrania and facial pain—no difference in the loins, thigh, and leg. Eight leeches behind the right ear—the same number to the loin—and a purgative of sulphate of magnesia. In ten days more the whole of the symptoms had gradually disappeared.

Dr. Martinet comes to the conclusion, that these phenomena could not have depended on affection of the brain, but on neuralgia. "The same cause," says he, "which exalted the sensibility of the sciatic, lumbar, and facial nerves, determined paralysis of the small branches going

* Revue Medicale, Janvier, 1824.

to their respective muscles." The absence of paralysis in the upper extremity is also in favour of our author's pathology; for where the brain is in fault, the paralysis usually shews itself first in the thoracic members.

*Case* 2. Louis-Joseph, aged 59 years, had been subject to irregular attacks of pain in his head from the age of 30. He had also occasional fits of palpitation, augmented by work (he was a copper-founder) and various moral affections. In the month of September last his head-ache increased, and on the 25th he was seized with the following symptoms, while going up to his work-shop:—general shivering—increase of the cephalalgia—embarrassment of speech—sudden pain in the cheek, temple, arm, thorax, and lower extremity of the left side, with sense of formication and considerable diminution of muscular power, so that he was obliged to be carried back to his apartment. In a few days these symptoms so far subsided that he tried to walk out a little, but was soon visited with a return of all the symptoms above-described. On the 1st of October he entered the Hotel Dieu. Here the symptoms continued for several days, but gradually diminished, and at length entirely disappeared, under mild diaphoretics, and the application of fumigations to the side affected. M. Recamier, under whom the patient was, did not hesitate to consider it as neuralgia, having seen several other cases of a similar description, all of which got well by fumigations. We would only just remark that, when the symptoms are equivocal at first, the safest plan would be to act as if the brain were the organ in fault. Evacuations can do little harm, for a while at least, in neuralgia, but the neglect of them might be of the worst consequence in cerebral affection inducing paralytic symptoms.

---

4. *Inflammation of the Semilunar Ganglia.* Professor Lobstein has written largely on the structure, use, and diseases of the great sympathetic; an excellent analysis of which work is given in the February and March numbers of our respected cotemporary, the Medical and Physical Journal. From the pathological portion we shall make some extracts.

Among the diseases connected with, or dependent on, morbid conditions of the great sympathetic, Professor L. ranks hypochondriasis, hysteria, melancholia, mania, angina pectoris, asthma, &c. Primary disorder of the ganglionic system will also induce, sympathetically, hemicrania, tinnitus aurium, and various other affections. We shall now extract a few cases where dissection came to the aid of observation during life.

*Case* 1. A female had suffered from spasmodic and hypochondriacal symptoms from the age of puberty, and had had two incomplete apoplectic seizures, with slight subsequent paralysis. She got married, and became pregnant. In the 8th week of utero-gestation a most obstinate and severe gastric irritability came on, in consequence of which she could keep no food whatever on her stomach for the ensuing three months, at the end of which she died. The internal fauces and mouth became inflamed, and ultimately mortified, from the constant vomiting. But the most distressing symptom was a burning pain along the course of the spine, and in the lower part of the right hypochondrium, producing continual jactitation, exhausting the patient's strength. The lady died at the end of the fifth month of pregnancy—not having received any relief from medicine. An attempt was made to produce abortion, but failed.

*Dissection.* The brain was sound, as were also the thoracic viscera. The stomach presented no morbid appearance, although there had been black vomiting. No disease could be discovered in the intestines nor in the uterine system, except some fibrous tumours in the substance of the womb, the size of walnuts. The fœtus was healthy, and in its natural position. The viscera were all removed, and the semilunar ganglia cut out. "It was not, indeed, converted into a foreign substance, but its colour was intensely red, which was regarded as true and genuine inflammation, by some men of experience and great cultivators of anatomy, to whom I communicated this observation. The inflammation was so obstinate, that the ganglia were but little paler after three days' infusion in cold water; and I took care to have the right ganglion drawn in this state, and represented in the seventh plate of this treatise. Its upper part is of a vivid red; but the inferior, from whence the mesenteric branches go off, is more livid. The splanchnic nerve appeared to me much broader than usual before its entry into the ganglion." The author conjectures that this inflammation had been long present, in a chronic form, giving rise to the hypochondriacal affections above alluded to. To this inflammation he also attributes the burning pain along the spine. The spinal cord does not appear to have been examined.

*Case 2.* The body of a girl was examined, who had been attacked with epidemic cough, which was converted by metastasis, first into spasmodic vomiting, and then into convulsions that could not be overcome by any remedial agents. In this case the left portion of the solar plexus was inflamed. The part was steeped for some days in water, at the end of which the redness had passed into a yellowish tinge, confined to the portion supposed to be inflamed.

---

5. *Cardiac Disease.** Desjardins, 22 years of age, of feeble constitution, had been, for a good while, affected with shortness of breath, especially if he attempted to run. In the middle of July 1821, he was suddenly seized, without apparent cause, with pains in his limbs and dry cough. Next month the glands of his neck swelled considerably—the cough continued; but he pursued his avocations till the 25th of September, when he presented himself at the Hotel Dieu, with the following symptoms:—pallid countenance—lips puffed and bluish—cough troublesome—expectoration mucous and ropy—breathing very short, difficult, and almost impossible in the recumbent posture; he

---

* M. Breschet. Archives Generales, Dec. 1823.

was therefore obliged to be always sitting up in bed, the body bending forward, and enjoying very little sleep. On percussion, the whole of the chest, but particularly the front of it, emitted a dull sound. He could take in but a very limited portion of air at each inspiration. The pulse was small and quick—the action of the heart imperceptible—heat of skin natural—no distinct pain in the chest. Twenty leeches were applied to the right side of the thorax. On the 18th the same number were again applied. On the 22d the same symptoms continued—two blisters in succession. The following days presented some amelioration of the symptoms, inasmuch as the patient could lie lower down in bed. 5th November, symptoms as bad as ever. Seton introduced in the left side of the chest. A considerable hæmorrhage resulted, and continued 24 hours. On the sixth and eighth a nasal hæmorrhage took place, requiring the plugging of the nostrils. He died on the 13th of the same month.

*Dissection.* In the left cavity of the chest about six or eight ounces of sero-sanguineous effusion—pleura costalis et pulmonalis opake—thickened—and very much injected—recent adhesions on the right side between the lung and diaphragm, with red granulations on the diaphragmatic pleura—both lungs gorged at their lower parts. The pericardium was enormously enlarged, and thickened in its structure to an inch or an inch and a half in some places, resembling the substance of the uterus, and in some parts like cartilage. The heart was rather larger than natural.

---

6. *Gangrene of the Heart.* This is a very rare disease. Our able and indefatigable friend, Dr. Kennedy of Glasgow, has published a brief but instructive memoir on this subject, in the April number of the Medical Repository. The case which gave origin to this memoir was a woman little past the prime of life, and who, in July 1823, came under Dr. K's care for apparently uterine disease of some two or three years' standing. She suffered from obstinate constipation, strangury, offensive uterine discharge, tendency to fainting, pain in the head, and what was most particular, "an excessive morbid perspiration, which wasted her strength, depressed her intellectual energies, and superinduced despondency." This perspiration was offensive, and not unfrequently manifested a peculiar nature. About the lumbar and dorsal regions, it often imparted to her undress a pale sanguine tinge, and this appearance was always followed by a sense of profound exhaustion and irregular nervous agitations. By a judicious course of medicine, calculated to improve the state of the digestive organs and recruit the general health, this patient, in a few months, became greatly amended in all respects, when, on the 4th November of the abovementioned year, she exposed herself without due consideration, to cold, wet clothes, and immoderate fatigue. This was followed (on the 6th) by sharp pains through all parts of her body, and an intense rigor, succeeded by great exhaustion and restlessness. 8th, at noon, Mr. Thompson (by Dr. Kennedy's desire) abstracted about 20 ounces of blood from her arm, and exhibited purgatives and antimonials. By these means the thoracic symptoms were relieved, but an acute pain arose in the region of the liver, to which a large blister was applied, without effect of any kind. In the evening an exacerbation of all the symptoms. The blood was inflamed. It was not till the 10th that Dr. Kennedy himself saw the patient. He prescribed another venesection, and an aperient lavement. The quantity of blood obtained was trifling; but the enema brought away three dark and offensive motions, without any mitigation of the symptoms. 11th, Dr. Kennedy and Mr. Thompson met in consultation, and perceived the rapid wane of their patient's living powers. In an hour afterwards she expired. The history of the symptoms is not at all detailed as the case went on, but Dr. K. gives the following particulars after the patient's death.

"Her pulses, at first small, hard, irregular, rapid, afterwards became feeble and less frequent, and intermittent: long before death, they disappeared altogether from the wrists. Syncopal tendencies often recurred, but never terminated in fainting. A sensation of burning heat pervaded all the regions of her chest; in the left it was excruciating. During the last four days of her life, the patient suffered much from stinging pains, which first occupied her extremities, then pervaded the shoulders and line of the vertebral column, then seized the left thoracic department, and, after the last bleeding, went, to be immoveably fixed, in the right hypochondriac region. She had catchings in the chest and orthopnœal breathing.

"At an early period, cardiac palpitations supervened, and gradually acquired frequency as well as strength. As the disease advanced, however, and the powers of the heart began to yield, its actions became very intermissive and troubled. All the extremities, superior and inferior, suffered progressive tumefaction from the diffusion of extravasated lymph. The left arm and limb, in particular, were distended almost to bursting; their joints, at first nearly inflexible, could not, during the three days which preceded her dissolution, be moved without occasioning exquisite pain. Broad livid patches, in some places, disfigured their surfaces; on others, were large sphacelated wheals. At the same time, she was altogether so impotent as to be incapable of changing, in any degree, her position, without assistance. Many hours before her demise, she fell into a state of comatose abstraction, which uninterruptedly increased, till the last of her vital forces irreparably failed." 279.

*Dissection, 8 hours post mortem.* Some black patches on the omentum—mesenteric vessels much distended—many spots of inflammation and others of gangrene on the intestines. Uterus twice its natural size—external surface of the fundus soft and livid. Liver of a dark colour, but not disorganized in substance. In the thorax twenty ounces of turbid serum were found—and four ounces of a similar fluid in the pericardium, on the internal surface of which was much vascular network of a dark colour.

"All parts of the heart, external and internal, exhibited distinct marks of having been the seat of gangrenous inflammation. They were preternaturally flaccid, and dark in colour as the darkest coagulated venous blood: they could be easily perforated in every direction with the finger. When thus torn, they exhaled a putrid odour; but no blood exuded from their ruptured vessels. The left ventricle, in particular, was quite livid, and destitute of its muscular tenacity; it was little firmer than cerebral structure. When lacerated, it threw out a most offensive odour, not different from what is generated by putrescent animal substance. All the cavities of the heart were empty; but the large veins, especially the abdominal, were loaded with grumous blood." 280.

"Though heavily loaded with blood, the lungs appeared healthy. Patches of morbid structure, unequal in size, and discoloured to various shades of darkness, had formed on different parts of the surface of the body—some of them putrescent, others putrid."

It appears from the dissection, that a remarkable tendency to gangrene existed not merely in the heart, but in various other organs and structures of the body of this female. For various interesting comments we must refer to the original paper in the Repository.

---

7. *Scirrhous Pylorus.* Dr. Abercrombie has favoured the profession with some cases of, and observations on, disease of the pylorus. This affection is, of course, beyond the reach of medicine. It is often obscure in its symptoms, but generally accompanied by dyspepsia, obtuse pain, especially after eating—sometimes acute and violent attacks of pain and vomiting, with considerable intervals of ease. These intervals often deceive practitioners, inducing them to believe that no serious organic affection can be present in a viscus like the stomach, with days and weeks of ease and apparent integrity of function. We lately attended a patient, who had been affected with scirrhous pylorus for years, and yet the paroxysms of pain seldom at first lasted more than one, two, or three days, attended with vomiting, after which an interval of from two weeks to two or three months would succeed, during which, digestion went on well, and the system would recruit from the effects of the preceding attack. In progress of time the paroxysms became more violent, the intervals of ease shorter, and the recovery less perfect. Emaciation ultimately advanced, and the patient died, worn out with pain, and deprived of nutrition. The following case recorded by Dr. Abercrombie forms an appropriate illustration.

"A gentleman, aged 30, was for several years liable to paroxysms of pain in the stomach. The pain usually continued for several hours, and went off with vomiting, and it returned at uncertain intervals, frequently of many weeks. Upon several occasions, he appeared to have got entirely free from it. He was in other respects in good health, except being considerably troubled with hæmorrhoids, until about a year before his death, when he was suddenly seized with copious vomiting of blood. This soon went off, and in a few days he was in his usual health; but from this time his former complaints rather increased. The attacks of pain, followed by vomiting, were more frequent, and he was again several times attacked with vomiting of blood, but still he had considerable intervals of health; no hardness could be discovered by examination, and that uniformity of symptoms was entirely wanting which usually accompanies organic disease. His death was rather unexpected. After having complained for two days of pain in his stomach in the usual form, he was found, in the morning of the third day, exhausted and without pulse, and died in a few hours. Three days before his death, he had been able to walk out a good deal, and made no particular complaint.

*Dissection.*—The pylorus was surrounded by a mass of scirrhus, the size of an orange, very firm, nearly cartilaginous. Among the intestines there were extensive adhesions. The body of the stomach, the liver, spleen, and pancreas, were healthy. There was some ossification of the valves on the right side of the heart, and the right auricle was remarkably distended with coagulated blood. The lungs were healthy." 244.

Dr. A. relates another case still more remarkable, from its progress, and the absence of all the symptoms which usually are looked for in organic diseases of the pylorus.

*Case.* A man, aged 40, came under Dr. A's care in December 1817, weakened and emaciated, with pulse 120, but without any other complaint. No pain, no cough—appetite good, bowels natural, gastric functions healthy. A hardness was felt about half way between the ensiform cartilage and the umbilicus, painful on pressure. He had been ill 18 months, the affection having commenced with vomiting, which recurred five or six times a day. This state continued five or six months, and then ceased entirely. During the last twelve months he complained of nothing but debility and emaciation. He died completely exhausted, and without any other symptom supervening except violent fits of pain in the abdomen. There was no vomiting, and the appetite was tolerably good till the last. On dissection, a large mass of scirrhus, four or five inches in extent, surrounded the pylorus; and the pyloric orifice was so narrow as scarcely to admit the point of a very small finger. The inside of the mass opened upon the inner surface of the stomach, by an ulcerated surface, covered with large cancer-looking tubercles. The rest of the stomach was sound.

There is perhaps no other organ in the body where disease will arrive at a greater height with fewer symptoms than the stomach—notwithstanding the delicacy and importance of the viscus. The reader will remember the case of Napoleon Buonaparte as a memorable example.

8. *Post-mortem Appearances in Hooping Cough.* Dr. Webster has drawn the attention of the profession, as our readers know, to the pathology of hooping-cough, which he seems to consider as kept up, if not caused, by vascular turgescence in the head. The only fatal case of the disease which has lately happened in our author's practice was examined by him after death. The child was only nine months old, and had had the disease one month before it proved fatal. When first seen by Dr. Webster, in the middle of the above period, the child appeared to suffer greatly in the head, in addition to the hooping-cough, which recurred every five or ten minutes. The eyes were red and heavy, the head hot, the pulse quick, the constitutional fever considerable. Leeches to the head, and aperients gave temporary relief; but in 14 days from admission, "the patient died, having *every symptom of hydrocephalus*, with occasional fits of hooping, but not so severe as at first."

Here it is evident that the child did not die of hooping-cough, and therefore we could not reasonably expect the same appearances in the chest which present themselves in fatal cases of that disease. The external aspect of the lungs was healthy—they were crepitous throughout—neither was there any disease on the mucous lining of the air-passages, except at the bifurcation of the trachea, where there was a slight blush of redness and some frothy mucus. In the membranes of the brain there was great vascularity, and a good deal of serous fluid effused between them. In the ventricles about two ounces of serum were found, and their arachnoid lining was injected with blood-vessels.

In the above case the symptoms and dissection prove that death was caused by the cerebral disease, but whether that disease was a mere coincidence or consequence of the hooping-cough, is not proved. Allowing, what is very probable, that the cerebral affection was caused by the interrupted return of blood from the head, in consequence of the cough. There is nothing in this case to support the idea that the cause of hooping-cough is in the head. To the treatment recommended by Dr. Webster, and employed by him with so much success, we do not object, but, on the contrary, highly approve. This consisted in the application of leeches to the forehead and behind the ears, blisters, and occasional purgatives. It is clear that this treatment guards the brain from congestion, so liable to take place in most diseases of children. The hooping-cough itself will generally do well, under any common antiphlogistic methodus medendi.

---

9. *Cases of Disease of the Heart, from Contraction of the left Auriculo-Ventricular Opening.* By M. Bouillaud, M. D.*

The science of diagnosis has made great progress within the present century; and in no diseases more than in those of the heart. "Thanks," says Dr. B. "to the important instrument discovered by Laennec—thanks to auscultation, mediate or immediate, any student, who has studied the use of the stethoscope, can now recognize by easy and certain signs, the narrowings (retrecissemens) of the auriculo-ventricular openings of the heart." These contractions derange, of course, the valvular apparatus placed between the chambers of the organ, and give rise to the most serious diseases. It is found by experience, that the *left* auriculo-ventricular opening and the aortic opening are much more frequently affected by induration and contraction than the *right* auriculo-ventricular open-

* Archives Generales, September 1823.

ing; the reason of which is not satisfactorily accounted for by the difference between arterial and venous blood circulating through them.

Corvisart's work furnishes us with no positive signs by which we can ascertain contraction of the *right* auriculo-ventricular opening—because, says he, we have no means of feeling the pulmonary artery. There is, however, he observes, a peculiar murmuring noise in the chest, in these cases, which it is very difficult to describe. The pulse is not so irregular as in auriculo-ventricular contractions of the *left* side. It is in contraction of the aortic orifice and induration of the semilunar valves that the pulse is remarkably affected. "The pulse, in this last case," says he, "may still preserve some degree of hardness, but is never either full or regular. This permanent irregularity of pulse is a sure diagnostic sign of contraction of the aortic opening." It is by mediate or immediate auscultation, however, as laid down in the admirable work of Laennec, that we are to form our diagnosis in these diseases. We think every physician should possess himself of, and carefully study the work alluded to—it is one of infinite importance in the diagnostic, and consequently prognostic branches of our science.

*Case* 1. Lebaut, 68 years of age, a robust female, but with ill-formed chest, entered the hospital Cochin, on the 4th November 1822. She reported that she had been in the habit of *vomiting* blood for five years past. During the last three years she had laboured under symptoms of aneurism of the heart. Carefully examined, she presented the following phenomena:—cough—sense of constriction in the middle of the chest—palpitation—orthopnœa—fear of suffocation—violet hue of countenance—lips swelled—pulsation of the jugular veins—pulse irregular, unequal, intermittent, frequent, and small—pulsation in the region of the heart strong. When the stethoscope or naked ear was applied to the chest, the ventricular contractions were found to be intermittent and irregular—the intermissions, in general, being preceded by two quick contractions of the ventricle. The contraction of the left ventricle was accompanied by a strong impulsion, and peculiar noise or susurrus. The hand applied to the region of the heart felt a vibratory tremor, deep-seated but very distinct. The lower extremities were œdematous. *Diagnosis* recorded on the books was, *contraction of the left auriculo-ventricular opening—hypertrophy and dilatation of the left ventricle.* She died eleven days after entrance into the hospital.

*Dissection.* The heart enormously distended, with clots of blood, and nearly three times its natural size. The left ventricle was dilated, and its parietes about an inch in thickness towards the base of the heart. The carneæ columnæ of the mitral valve were very strong. The right ventricle was a little thicker in its parietes than natural, but not sensibly dilated. The two auricles were dilated, and thickened in their parietes at the same time—the left being much more so than the right. The substance of the heart was firm and red. The mitral valve was disfigured, hard, and fibro-cartilaginous. The left auriculo-ventricular orifice was so contracted that the point of the little finger could hardly be pushed through it. The tricuspid valve was deformed also, but the right auriculo-ventricular orifice was very large, and could not be closed by the valve. The pericardium reflected over the heart, presented a white patch of false membrane, and a number of small warty excrescences. The semilunar aortic valves were thickened, but the orifice of the aorta was not sensibly contracted. We need not enumerate the appearances in the other parts of the body, as the patient evidently died of disease of the heart.

*Case* 2. Louisa Newray, aged 33 years, had not menstruated during the last six months, and being affected with much embarrassment about the chest, she determined to enter the hospital, on the 22d of September, 1822. Reported, that eight years previously she experienced a fall on the præcordial region, since which she had palpitations, orthopnœa, and spitting of blood. She was under the care of Professor Pelleton, in the Hotel Dieu, and by repeated small bleedings and low regimen her condition was so much improved, that she left the hospital at the end of five months in tolerable health, excepting that she experienced, from time to time, especially on taking any exercise, palpitations, and sometimes orthopnœa. When received into the Cochin, she presented the following phenomena;—palpitations very strong—and stronger to her own feelings towards the right than towards the left præcordial region—paleness of countenance—air of suffering and anxiety—protuberance of eye, and expression of fear there—lips vermillioned—oppression, orthopnœa, cough, sanguineous expectoration, wheezing respiration, small, hard, and quick pulse, but regular. The cardiac pulsations could be felt by the hand to a considerable distance around the point where the organ usually beats. These pulsations were much more apparent in the situation of the right, than of the left ventricle.

*Auscultation.* The pulsations of the right ventricle are accompanied by a strong impulsion, and resemble the strokes of a hammer—they are heard in the posterior part of the chest. The beating of the left ventricle presents nothing extraordinary—pulmonary rattling very sonorous.

*Diagnosis.* Aneurismal hypertrophy of the right ventricle—contraction of the left auriculo-ventricular orifice—bronchial catarrh.

By bleedings, quietude, and diluents, the patient so far recovered as to be able to leave the hospital in a month; but, as might be expected, she returned again in six weeks, worse than ever. She had now discharges of blood from the lungs, constant sense of suffocation—inability to lie down—sleep interrupted by frightful dreams and startings—in short, the situation of this poor woman was dreadful. The pulsations of the right ventricle could be felt in every part of the thorax—even by the hand quite back near the spine. The pulse was small, but perfectly regular. She lingered out a wretched existence of better than a month after her second entry into the hospital, and then expired suddenly.

*Dissection.* Considerable dropsical effusion into the cellular membrane of the lower extremities, but very little in the bags of the pleura—lungs voluminous and crepitous—lining membrane of the bronchia red—heart full of clots of blood and of enormous volume—when these clots were removed, it was still considerably larger than natural—the cavity of the right ventricle much dilated, and its parietes about half an inch in thickness, being of a red colour, and denser than natural—orifice of the auriculo-ventricular communication natural—right auricle dilated in proportion to the ventricle; its parietes thicker and more fleshy than natural—left ventricle natural, and its parietes about the same thickness as those of the right chamber—the mitral valve thickened and indurated—the auriculo-ventricular opening in the left side contracted and elliptical, so as scarcely to admit the point of the little finger.

*Case* 3. Mary Simon, aged 47 years, had ceased to menstruate five years. She entered the hospital Cochin on the 21st of February, 1822. She had experienced much domestic affliction and chagrin. In 1813 she began to perceive that her hands and face were changing to a violet colour, that her lower extremities swelled, and that she had palpitation and dyspnœa on the least exertion. These symptoms were relieved at the time by aperients and diuretics; but some degree of palpitation continued. In 1817, the increase of the abovementioned symptoms, together with the addition of hæmoptysis, forced her to enter the Hotel Dieu, where she was under the able care of M. Recamier. After this she had been in the Cochin Hospital, and received temporary relief; but still the symptoms continued, aggravated at intervals. When she came under the care of the reporter in the hospital Cochin, for the last time, she had hæmoptysis—livor of countenance and skin of the extremities—pulse hurried, irregular, intermittent, very small, contrasting greatly with the violent palpitation in the region of the heart, which raised the sternum at each shock—pain in the left side of the chest—great sense of suffocation.

*Auscultation.* The pulsations of the heart were so violent and tumultuous that an analysis of them was very difficult. Those of the left ventricle communicated to the stethoscope a strong impulsion, and were moderately sonorous—in the region of the right ventricle there was heard a hissing noise—mucous rattling in the bronchia very distinct in the anterior part of the chest—*pectoriloquism* distinct under the right scapula.

*Diagnostic.* Contraction of one of the orifices in the left side of the heart with hypertrophy—tubercles in the lungs with excavations. Died on the sixth day after entering the hospital.

*Dissection.* Adhesions of the pleura very general—a portion of the left lung tuberculous—upper portion of right lung tuberculated and excavated—the rest of the lung sound—heart nearly double its natural size, and pushed high up in the chest by the abdominal organs—*left* ventricle voluminous, its parietes about an inch in thickness, but its cavity not much increased in dimensions—left auricle rather thickened in its parietes, and the carneæ columnæ of *its* appendix so large that they resembled those of the ventricles—left auriculo-ventricular orifice contracted greatly—bicus (mitral) valve indurated and deformed—the *right* auricle and ventricle filled with blood, but not beyond the medium size—the ventriculo-aortic orifice was puckered and contracted in consequence of three roundish tubercles growing from the three semi-lunar valves—some cartilaginous patches on the inner membrane of the aorta. In the abdomen, it was remarked that the organs were somewhat large, but very much incroaching on the thorax, being pushed up there apparently by the strong action of the abdominal muscles.

*Case* 4. Ellen Lemindre, 34 years of age, having experienced much domestic trouble, began to feel some symptoms indicative of disease of the heart in the course of the year 1820. In the year 1821 she spit up some blood—she had considerable oppression in the chest, with palpitation after the least exercise. She entered the Cochin on the 7th of February, 1822, presenting the following phenomena—suppression of the menses for two months—face swelled, but not livid—œdema of the lower extremities—pain in the chest, especially in the præcordial region—sense of oppression there on making the least effort—slight orthopnœa—frequent cough—muco-sanguineous expectoration.

*Auscultation.* Applied over the left ventricle, the cylinder conveyed a very remarkable susurrus to the ear—the ventricular contractions very strong, while the pulse was very small but hard—the susurrus preceded the ventricular contractions, and consequently took place during the action of the left auricule—the region of the right ventricle and auricle presented nothing extraordinary—the *râle* (noise of air passing through mucus in the lungs) sufficiently audible.

*Diagnosis.* Contraction of the *left* auriculo-ventricular orifice, with hypertrophy of the left ventricle—pulmonary catarrh. The patient got a little better by repose, low living, and depletion; but was seized with erysipelas of the face and neck, which, with the original disease, put an end to her existence about three weeks after her entrance into the hospital.

*Dissection.* The lungs crepitous throughout, but the mucous membrane of the bronchia was red and inflamed—the heart was much larger than natural, and filled with blood in clots—parietes of the left ventricle full an inch in thickness, and its cavity rather diminished—left auricle dilated and thickened in its parietes—the left auriculo-ventricular orifice greatly contracted and puckered, so as to be only a few lines in diameter—the mitral valve misshapen, and of a fibro-cartilaginous hardness. Some deviations from natural structure in the right side of the heart, but not of material consequence.

A pretty general opinion prevails among medical men, especially of the old school, that all assistance from auscultation in diagnosis of cardiac diseases, is useless or unnecessary, as the general symptoms are quite sufficient indications of the nature of the malady. They are no such thing. These practitioners, if taken to a patient labouring under organic disease of the heart, will give a pretty good guess that the organ in question is diseased—but, if you ask them what is the *kind of lesion*, they *shake* their heads, and tell you it is impossible to ascertain that point. Thus, then, they can form no *diagnosis* at all, and consequently the

*prognosis* is a mere hap-hazard guess, which, fortunately for their reputation, generally turns out right in the end, because all diseases of the heart are dangerous, and most of them fatal. But there is great diversity in the organic lesions of this viscus, requiring considerable modification of treatment, and consequently requiring accurate diagnosis, if it can possibly be attained. On this account we should not disdain any help which auscultation (mediate or immediate) can afford. Functional diseases of this organ are very apt to imitate very perfectly the phenomena of organic diseases—and here the practitioner who trusts solely to general symptoms, is almost always wrong, both in diagnosis and prognosis, to the great danger of his professional character, in these days of scrutiny and investigation prevalent among the rising generation of the profession. We seriously warn those, who think themselves very comfortably fortified in the old routine of experience, to bestir themselves in the task of keeping pace with the progress of knowledge. We have much to learn, as well as to unlearn.

---

10. *Neuralgia.* Dr. R. Evans has related two cases of neuralgia cured by carbonate of iron, in the last number of our respected northern cotemporary. The first was that of a gentleman who had been troubled for many years with a pulsating sensation in the base of the brain, which was ascribed by his medical attendants to an aneurismal or other organic affection. This continues, without disturbing his health. But the neuralgia was seated in the branches or twigs of the ophthalmic branch of the fifth pair of nerves of the right side, occupying, at first, a circular spot the size of a dollar, and about 2½ inches above the supra-orbital foramen. The pain was not constant during the day, but was always excited by the most trivial touch of the finger or other external agent—even a slight current of air. The paroxysms became so violent, at last, that he could scarcely take food. When quiet in bed the pain went off. This state had continued 18 months. Opium and other narcotics were used without success, and the digestive organs were *Abernethianized* without any benefit to the local complaint. Under the exhibition of cinchona rubra, the seat of the pain shifted to the temple, but there it was as troublesome as before. Leeches dislodged the pain from this part, and it fixed itself in the trunk of the nerve as it leaves the supra-orbital foramen. After various remedies had been tried in vain, the carbonate of iron was exhibited in doses of one drachm thrice a day. In a few days Dr. Evans was surprised to find the disease nearly cured. The medicine was continued for some time longer, attending to the state of the patient's bowels, when the cure was completed.

The second case was that of a female, aged 45 years, who had been afflicted with neuralgia of the superior maxillary nerve (affecting all the teeth supplied by it, and the side of the nose) for the space of three years, the disease occurring in paroxysms, with very short intervals. She could only take food in a liquid form, and got very little sleep. She was put on a course of carbonate of iron (half a drachm thrice a day), and in between two and three weeks she was perfectly well.

Immediately succeeding Dr. Evans's cases, is one from Dr. Borthwick of Edinburgh. This was a man, aged 46, who had had a rheumatic affection of the right hip the preceding year, of short duration. Soon afterwards he was seized with a severe pain in the left side of the face, coming on in paroxysms which generally ended in a pain in one of the teeth of that side. Various remedies were tried in the country, and some of the teeth were extracted, without benefit. He came up to Edinburgh, and, in a consultation with Dr. George Wood, it was determined on to give the carbonate of iron, commencing with half drachm doses thrice a day, keeping the bowels open with gentle medicines. In five or six days the disease was subdued, and the patient continued well afterwards.

---

10. *Inoculation of Syphilis.* The new physiological doctrine of Broussais admits not the existence of specific poisons, specific diseases, or specific remedies—and why?—because they cannot be explained on the new principles!—To prove their non-existence, three young Parisian students inoculated themselves with the matter of a venereal chancre, in the month of November last. In one, a swelling of the axillary glands followed the introduction of the virus, and terminated in suppuration, with considerable loss of parts. The second exhibited several unequivocal symptoms of syphilitic infection. In the third, the puncture inflamed and ulcerated—a chancre, displaying the regular syphilitic character, appeared, and was treated by antiphlogistics, the young sceptic carefully abstaining from all mercurial preparations. The ulceration made fearful progress, but the youth obstinately refused to take the *specific.* At length, he consulted one of the professors of the *Faculté de Medecine,* who pronounced the ulcer to be syphilitic, and incurable without mercury. The young man, on hearing this sentence, returned to the hospital, and preferring death to a retraction of his preconceived opinion, cut the crural artery and died on the spot!

---

12. *Memoir on the Pathological Anatomy of the Peritoneum.* By M. Scoutteten, M. D. attached to the Military Hospital of Toulouse.

[Archives Generales, December, and February.]

Immediately after inflammations of the mucous surfaces come (in point of frequency) phlogoses of the serous membranes. These last too are more dangerous than the former. Schenck, Bonetus, and Morgagni thought that peritoneal inflammation was always *consequent* on inflammation of some other parts—and this has been maintained even by M. Portal. There are few, in the present day, however, who will deny that the peritoneum may be *alone* inflamed—and that very frequently. The writings of Broussais, Laennec, Gasc, Jeunesse, and others, in France, together with the various works on puerperal fever in this country, have thrown great light on the disease in question, and rendered the pathology pretty familiar to most of the observant part of the profession; but there is a great deal yet to be learnt respecting the chronic forms especially of peritonitis.

Our author divides his subject into three heads, 1mo. Those alterations of structure in the peritoneum dependent on inflammation—2ndo. Those structural changes which do *not* depend on inflammation—and 3tio. Foreign bodies in the cavity of the peritoneum.

1. *Peritonitis Acuta.* Our author thinks, and with justice, that it is needless to give various names to peritonitis, according to the portion of peritoneum which is inflamed, as omentitis, mesenteritis, &c. It is important, no doubt, to ascertain as nearly as possible, in what portion of the membrane the focus of inflammation is placed—merely that the external means of relief may be there applied—but to give names to these is worse than useless.

When irritation has been determined to the peritoneal membrane, and there produced the mildest shade of inflammation, we observe small red spots, not more than a line in diameter, separated from each other, and, on minute examination, appearing to be clusters of puncta crowded close together. Between these red spots, the peritoneum, when viewed with a good magnifying glass, presents portions of its surface of a natural colour. The appearance now described is rarely to be seen in man, for obvious reasons; but it may be readily produced in animals, as dogs, by injecting an irritating fluid into the cavity of the peritoneum. Thus, if bile be thrown in and the wound closed, the above appearances will be strikingly produced in 24 hours.

It has been remarked by several accurate observers, that all the symptoms of peritoneal inflammation have been present during life, and yet on dissection no trace of it could be found on that membrane. On this point our author professes to be sceptical. But we think there can be no reason to doubt that mere injection of vessels, or the first stage of inflammation, where the structure of the tissue is not altered, may disappear in the interval between death and dissection. Indeed, our author's own experiments give the greatest support to this opinion. Into the peritoneal cavities of dogs he injected bile, and at the end of 24 hours examined the abdomen, when inflammation was unequivocal. The animal was then immediately killed by pithing, and an evident diminution of the inflammation took place with death. As the dogs got cold, the diminution was still greater. These experiments were repeated many times, and always with the same result. It is therefore highly probable, if not quite certain, that the first stage of peritoneal inflammation, in some particular constitutions, may produce such disturbance in the vital functions as to destroy life—and that the collapse after death may dissipate the redness and other marks of phlogosis which existed anterior to that event.

In the early stages of inflammation, our author found by experiment, that the surface of the peritoneum, though apparently dry and glistening, was, when touched with the finger, covered with an unctuous and viscid exudation. Sometimes instead of the red spots above described, this first shade of phlogosis presented merely a development of red vessels running in lines to a greater or less extent.

In the progress of the inflammation the red spots become more extended and close together, ultimately so blended as to appear one homogeneous patch, of a scarlet colour. Still, at this period, the distended vessels are visible, but the peritoneal tissue does not appear to be thickened. It has, however, by this time, lost its transparency. When the phlogosis is still more advanced, the redness becomes more intense and extended, sometimes occupying the whole peritoneal expansion; at others, bounded to the forms of bands or stripes traversing various portions of intestine, or only occupying the space where the intestines adhere to each other. The intense red colour, at this period, is not wholly owing to the injection of vessels, but to a sanguineous exudation which is diffused over the peritoneal surface adhering very tenaceously to it, and presenting a villous appearance. Even in this stage, the peritoneum will sometimes appear dry and shining; but more commonly we now find an effusion of a whitish fluid into the abdominal cavity.

Such an acute inflammation of the abdomen can only last, our author thinks, a few days—three or four, without death or a change for the better. The abdominal pain is generally very acute—the patient drawing up the thighs, or bending the body forwards to relax the abdominal muscles. Sometimes the pain is bounded to a single point, and then we generally find the inflammation also bounded to the same spot. On the 3d September last, our author opened a man who had felt acute pain in the right iliac region for two days prior to his death. He found, on dissection, the marks of intense inflammation of the peritoneum bounded to the appendix cæci. At times, the peritoneum covering the bladder will be the seat of phlogosis, and then the evacuation of the urine will be almost uniformly suspended, and the pain felt in the pelvis. The peritoneum covering the inferior surface of the diaphragm may be the sole seat of inflammation, and then we have almost constant hiccup. A soldier in the *Val de Grace* hospital experienced, for some days, an intense gastro-enteritis, which began to give way to the usual means, when all at once a violent pain was experienced (augmented by pressure) in the direction of the diaphragm, accompanied by constant hiccup, shrinking of the features, drawing up of the legs and thighs. These symptoms could not be controlled, and he sunk in two days. M. Broussais prognosticated the existence of sub-diaphragmatic inflammation of the peritoneum, which was completely verified by dissection.

In acute inflammations of the peritoneum, we have this membrane sometimes of a purple or even black colour, with strong adhesions of the folds of intestines together, without the intervention of a false membrane, which at other times, is occasionally, indeed often, seen. Actual gangrene of this membrane has been found after death; but this is of rare occurrence.

Another phenomenon of still rarer occurrence presented itself to our author in the year 1822, in the body of a man who had died of acute peritonitis. This was a subperitoneal emphysema. The whole sheet of this membrane was equally elevated by gas, which could be pressed from place to place, and made to accumulate in particular positions. In another case he found the same phenomenon, but on a much more limited scale, the emphysema being bounded to the peritoneum lining the diaphragm and covering the liver. In neither of these instances was there any sign of putrefaction to account for the coalition of gas.

Between the laminæ of peritoneum forming the mesentery, we sometimes find collections of purulent matter, in peritoneal inflammation. This, however, is not of frequent occurrence.

When peritoneal inflammation is extended to 20, 25, or 30 days, false membranes of albuminous matter are found glueing the convolutions of the intestines together, and even these last to the peritoneum lining the abdominal parietes.

During the first few hours of acute peritonitis there is very little effusion of fluid beyond the usual halitus; but, after the inflammation has lasted 36 or 40 hours, there will be an evident effusion, generally of a whitish or milky appearance. Blood itself has been found extravasated in acute peritonitis—but this is a rare phenomenon. The quantity of effusion varies from a few ounces to some pints. Sometimes it is nearly as limpid as water and containing no albuminous flocculi;—in other cases, it is thick, and resembling diluted pus of a very peculiar odour, which can never be forgotten by those whose hands have been imbued in it.

II. *Alterations of Structure observed after Chronic Inflammation of the Peritoneum.* Chronic is often the consequence of acute peritonitis; but it also not unfrequently creeps on in a slow and almost insensible manner, without any violent symptom. This last may be properly termed "primitive chronic peritonitis."

*Chronic after Acute Peritonitis.* On opening the body of a person who has had peritonitis for 50 or 60 days, we shall find the abdomen containing a greater or lesser quantity of a whitish fluid—a number of false membranes glueing the intestines together, or forming sacs which contain fluids of different appearances. When these false membranes are detached from the peritoneum we shall find that structure less red than in acute peritonitis; sometimes indeed scarcely coloured. In these cases the effused fluid is rarely in such quantity as to sensibly distend the parietes of the abdomen. In some cases, however, a considerable quantity of limpid yellow serum will be found, without any trace of false membranes, but with the peritoneum thickened, reddish, and highly injected—or the great omentum thickened, red, fleshy, or presenting hydatiform bodies. These two shades of peritonitis are not attended with much pain, and bear abdominal compression without much inconvenience. The patients only complain of a sense of weight, and they are much harrassed with constipation of the bowels. When chronic peritonitis has lasted many months, in certain constitutions, we find, besides numerous adhesions and false membranes, a development of tubercles, of different sizes, and various degrees of consistency.

Instead of the serous, or puriform fluids which we generally find after peritoneal inflammations, there is sometimes, though rarely, an extravasation of sanguinolent, or even pure sanguineous fluid, resulting from rupture of vessels.

Of ulceration, gangrene, and scirrhus of the peritoneum, we shall not speak; as they are extremely rare in their occurrence.

The above are the general appearances presented after inflammation, acute and chronic, of this important membrane, and the practitioner should bear them in mind when he is prosecuting his post mortem researches.

---

13. *Angina Pectoris.* Dr. Michael Ryan has published a case of (supposed) angina pectoris cured by prussic acid. We shall state the case before we offer our comments on it.

Mary Lalor, ætat. 21, thin and delicate, applied to our author on the 8th July 1823, on account of pain under the left mamma, suddenly shooting across to the sternum, on the slightest exertion, and inducing a distressing sense of suffocation threatening instant death and obliging her to stop. She had also a dull pain in the left shoulder extending to the middle of the arm, and sometimes to the fingers. Palpitations were troublesome, especially on ascending an eminence. These symptoms were of three years' duration—"*were mostly present every day*," and latterly had been attended with *dyspepsy*. The urine was scanty and the ancles œdematous—pulse 120, small and regular. Leeches and blisters had been previously applied, and many internal medicines exhibited, with only temporary advantage. The dyspepsia was relieved by the ordinary remedies, but the pain in the side and sternum remained. Dr. Ryan applied the tartar emetic ointment, and exhibited a mixture composed of digitalis, colchicum, and prussic acid. By these remedies she was soon entirely cured.

Dr. Ryan has little hesitation "in asserting that the affection of the heart was idiopathic, and what is called *angina pectoris;* while that of the stomach was only symptomatic of the former." A little farther on he observes that—"Angina pectoris is an *organic* disease of the coronary arteries of the heart, inducing a deficiency of nutriment in its parietes." Very well. On what principle did he administer the triple compound of digitalis, colchicum, and prussic acid?—This is his answer. "The digitalis was exhibited to regulate the circulation; the colchicum on account of the symptoms of incipient hydrothorax; and the hydrocyanic acid, to allay the irritation of the organs of respiration and circulation." So then the "*deficiency of nutriment* in the parietes of the heart," resulting from ossification of the coronary arteries, was compensated for by those mild, nutritious, and cordial medicines, digitalis, colchicum, and prussic acid!—It is hardly necessary to say that we come to a conclusion the very reverse of that to which Dr. Ryan came—namely, that this said angina pectoris, so very easily cured, was *not* an idiopathic affection of the heart, but symptomatic of disorder in the digestive organs. Dr. Ryan appears to have taken great pains to study the *history* of angina pectoris. We can pretty confidently assure him that, when he sees a little more of the *true nature* of the disease, he will not be quite so dogmatical as to its pathology. When he comes to open a few victims to this disease, he will probably be inclined to relax a little as to ossification of the coronary arteries being the essence of the disease. He will find the symptoms of angina pectoris resulting from *various lesions* of that organ—and when they depend on ossification of the coronary arteries, he will not cure them with digitalis, colchicum, and prussic acid, so readily as he cured Miss Mary Lalor.—*Med. and Phys. Journal, No.* 303.

---

14. *Meningeal Apoplexy.** Lady D——e, 62 years of age, of full habit, had been out in her carriage on Tuesday, 10th December 1822, in very good health and spirits. She returned at 4 o'clock, and at 6 was discovered on the floor of the drawing room, insensible and rolling about. Sir Astley Cooper and Dr. Johnson were summoned, and Dr. Warren and Mr. Freeman arrived soon afterwards. When placed on a sofa, Lady D. kept in a state of jactitation, throwing about her arms, rolling her head, and sighing. She was insensible when spoken to, her pulse weak and unequal—skin cool—face pale—pupils obedient to the stimulus of light. A vein was opened in the arm, but only a few ounces of blood flowed. She now appeared to be dying, but rallied a little after an attempt to vomit. Ten grains of the sulphate of zinc, without effect. Mr. Freeman opened the temporal artery, and about 36 ounces of blood were drawn off. On speaking loudly to the patient she now evinced some sensibility, and answered a few questions though indistinctly, relapsing again into a state exactly resembling common sleep. Ten grains of calomel were, with difficulty, got down the throat, and an assafœtida enema administered—and five grains of calomel with a black draught were ordered every four hours afterwards, till the bowels should be relieved. No evacuation through the night. At nine next morning (11th) the pulse had got up, and sixteen ounces of blood were abstracted. The pulse now fell—the respiration became laborious—and stertor appeared for the first time. These symptoms, however, disappeared, in a considerable degree—the bowels became freely opened—so much sensibility returned that she answered several questions, the pulse becoming regular, forcible, and about 80 in the minute. Sinapisms having been applied the preceding evening, they now occasioned great distress, and it required several attendants to confine the patient's struggles and prevent her getting out of bed. *There was not therefore any paralysis present.* The sinapsisms removed, she became tranquil, and again fell into a soporose state. During the whole of this day the pupils were obedient to the light—there was no paralysis or drawing of the face—

---

* Dr. James Johnson, Med. and Phys. Journal, No. 303.

and the patient was able to swallow liquids. In the evening, the coma having become more marked, cupping-glasses were applied to the temples, and sixteen ounces of blood withdrawn, when the pulse began to flag. At this time so much sensibility returned that Lady D. answered several questions, knew some of her relations, and called them by name. Very soon after ten o'clock, however, the coma returned—the breathing became laborious and stertorous—the pulse irregular. The patient continued to move the head and also the extremities, till three o'clock in the morning of Thursday the 12th, (33 hours from the commencement of the attack) when all voluntary power, except that of deglutition appeared to be lost, and the patient lay in a comatose state till half-past 8 o'clock in the evening, or 51 hours from the beginning of the attack, when she expired.

*Dissection*, by Sir Astley Cooper and Mr. Freeman; Dr. Warren and Dr. Johnson being also present. The dura mater presented no unusual appearance. The arachnoid was somewhat thickened and opake, with a moderate effusion between it and the subjacent pia mater. The pia mater itself, wherever it extended—over the whole of the hemispheres, between them, among the convolutions, and over the base of the brain, medulla oblongata, and as far as could be seen of the spinal marrow, was infiltrated with black blood, resembling very much the tunica conjunctiva of the eye, when highly *blood-shot.* This state was very different from that of mere congestion of the vessels. No blood-vessels, whether veins or arteries, were distinguishable; but the texture of the pia mater seemed completely impregnated or infiltrated with blood, so as not only to change its natural appearance, but quite to obscure its vessels. Besides this general infiltration, or *blood-shot* condition of the pia mater, there was a considerable quantity of blood distinctly extravasated in small globular coagula on the inner surface of the membrane, varying in size from that of a pin's head to the size of a very small pea. Wherever the pia mater was stripped off the brain, these graniform extravasations rose with it, leaving the surface of the brain clean, and of its natural colour. The brain itself was something softer than natural, but exhibited no marks of increased vascularity—no red points when sliced with the scalpel. In the lateral ventricles there was about an ounce of very red serum—a small quantity in the other ventricles and at the base of the brain—the whole amounting to about two ounces. The pia mater covering the cerebellum was in precisely the same condition as that covering the cerebrum. The lining of the ventricles had a reddish blush, but nothing like the *blood-shot* appearance of the pia mater. In the basilary artery and in the circulus Willisii, there were some points of calcareous deposition; but no ruptured vessel was perceptible in the brain.

This dissection offers one of the most exquisite specimens of meningeal apoplexy, perhaps on record. In this case, there was no rupture of any particular vessel—no local accumulation of blood—no laceration or excavation of any portion of brain—no *partial* though considerable *general* pressure. The physiological phenomena were in strict accordance with the pathological condition. The *blood-shot* state of the pia mater, while it produced a uniform pressure over the whole of the encephalon, was sufficient to abolish, or at least suspend, the intellectual operations, without actually paralysing the muscular system, voluntary or anlomatic. It may be observed, that when the effects of this general pressure on the brain were mitigated by the abstraction of 36 ounces of blood, perception and intellectuality were so far restored that the patient answered questions with some degree of distinctness—a circumstance that was still more evident on the evening of the second day, after the abstraction of sixteen ounces of blood from the temples.

It is stated by M. Serres that *meningeal* apoplexy may be distinguished from *cerebral*, in the living body, by the absence of paralysis and the presence of sensibility to stimuli in the *former*;—while, in the *latter*, (cerebral) there will be some degree of paralysis in one or other side, or drawing of the mouth. There was no evidence of paralysis in Lady D's case—for the general cessation of voluntary *action* during the last 18 hours could not be called loss of power as in paralysis, but merely that state which precedes death in most diseases. The profound coma, too, would prevent the feeling of any external stimulus, and thus mask muscular power, if it did exist. So far this case corroborates the diagnostic criterion laid down by M. Serres. In one hundred cases of fatal apoplexy at La Pitié, which were carefully watched before death, and examined afterwards, 21 were uncomplicated with paralysis—and in none of these was there any laceration of the brain, or local extravasation of blood. Sixteen of these presented *serous* effusion—one serosanguineous in the ventricles—two with a similar effusion between the arachnoid and pia mater—and two without any effusion at all, but only a general turgescence of the vessels. In every one of the other, or 79 cases, where there was paralysis of one or more members, or drawing of the mouth to one side, there was found some local extravasation of blood, in some part of the brain.*

The case here detailed presents a very fine example of local determination (as it is called) to a single membrane in the head, the other structures being left, as it were, untouched. It has been called a *coup de sang* by Continental pathologists, which corresponds with *blood-shot*, the expression here applied, and which conveys a more accurate idea of the appearance than any other term.

### III.

### SURGERY.

1. *Lithotomy.*† Mr. Key, one of the most promising young surgeons of the celebrated school that gave birth to the present lateral ope-

* See Dr. Cooke's valuable work on Palsy, p. 702.

